# The Soldiers' Daughters' Home, Hampstead

**Published:** 1914-03-07

**Authors:** 


### THE SOLDIERS' DAUGHTERS' HOME, HAMPSTEAD.

### A Grave Scandal: Where are the Health Authorities?

Very serious criticisms are being brought against the sanitary condition of the Soldiers' Daughters' Home at Hampstead. This school was founded in 1855, and was in the first instance intended for the education and training as domestic servants of the daughters of soldiers killed in the Crimean War. It is partly maintained by contributions from various regiments, and is an object which should appeal to civilians as well as to the military. But both as a home and as a training school it is unsatisfactory. The original designers—acting according to the custom of their time—built handsome reception-rooms, but were weak on the subject of kitchen accommodation. In accordance with the troglodytic customs of our grandfathers, all domestic work is done in a basement, stone-floored, gloomy, and ill-lighted. We are not quite accurate in saying "all domestic work." The washing-up after meals is done in the dining-hall itself—hardly a method to be taught to girls who are intended for decent service. The cooking utensils are washed in an ill-lighted scullery—a circumstance which cannot tend to fastidious cleanliness. After the morning's work some thirty girls are expected to wash themselves in two basins set in a gloomy cell, and if they are personally clean it is something of a marvel. The sanitary arrangements are on a par with the domestic ones. Many of the children come from poor homes, but that is no reason for keeping them during all their school years in an environment which perpetuates all the worst features of poverty, and does not give the training which would fit them to occupy a decent position in after-life. Naturally the health of the children is not good. There have been 150 cases of throat trouble since last September, besides some cases of diphtheria. It is intolerable that such a condition of affairs should longer continue. The committee must take immediate steps to reconstruct the present buildings and make them up to date and suitable for their purpose or close them pending the formulation of a well-thought-out scheme for the removal of the institution to the country to enable it to be conducted on the cottage-home principle, with a central school and assembly hall. For either scheme money will be needed, but money will be forthcoming, no doubt, if the facts are faced and an adequate scheme is prepared and widely circulated. Anyway, the continuance of this home on its present basis is too grave a scandal to be permitted, on the ground of the welfare of the unfortunate inmates and in the interests of the health of the community. H.R.H. the Duke of Connaught is the president of this home, and the King, the Queen and Queen Alexandra are the patrons. Would that King George or Queen Mary could spare the time personally to inspect this home, for then the risks our soldiers' daughters have at present to run would, we are confident, be ended quickly.

